# Highly photostable wide-dynamic-range pH sensitive semiconducting polymer dots enabled by dendronizing the near-IR emitters†
†Electronic supplementary information (ESI) available: Experimental materials and methods, characterization of all of the compounds, and supplementary figures. See DOI: 10.1039/c7sc03448b



**DOI:** 10.1039/c7sc03448b

**Published:** 2017-09-04

**Authors:** L. Chen, L. Wu, J. Yu, C.-T. Kuo, T. Jian, I.-C. Wu, Y. Rong, D. T. Chiu

**Affiliations:** a Department of Chemistry , University of Washington , Seattle , Washington 98195 , USA . Email: chiu@uw.edu

## Abstract

One constraint of semiconducting polymer dots (Pdots), especially those with near-IR emission, is their low effective emitter ratio (∼1.5 mole percent), which limits their pH sensing performance. The other critical issue of existing Pdot-based pH sensors is their poor photostability. To address these issues, we developed a series of Pdots by dendronizing the squaraine-based pH responsive near-IR emitter, which is covalently incorporated into the polyfluorene (PFO) backbone. The fluorescence self-quenching of the NIR squaraine emitter was effectively suppressed at a high emitter concentration of 5 mole percent. Through controlling the individually incomplete energy transfer from the amorphous PFO donor to the blue β-phase PFO and NIR squaraine emitter, we obtained a ratiometric pH sensor with simultaneously improved pH sensitivity, brightness, and photostability. The Pdots showed a fast and reversible pH response over the whole biological pH range of 4.7 to 8.5. Intracellular pH mapping was successfully demonstrated using this ultra-bright and photostable Pdot-based pH indicator.

## Abstract 2

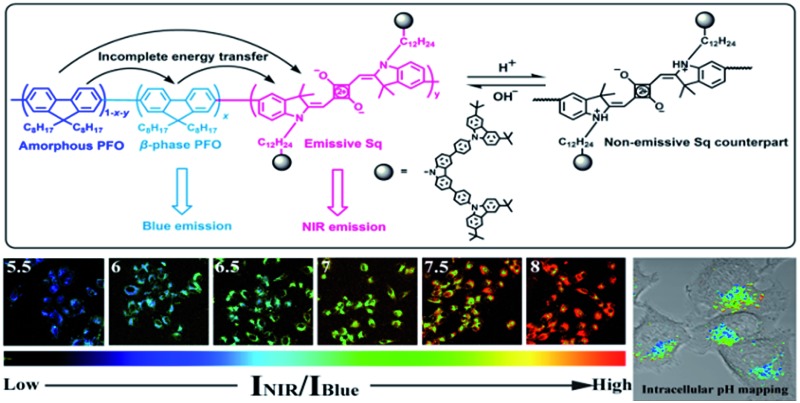
A highly photostable, ultra-bright, biocompatible and NIR emissive semiconducting polymer dot (Pdot) based pH sensor was achieved by dendronizing the squaraine probe molecule.

## Introduction

Intracellular pH (pH_i_) sensing and mapping is of great interest and importance for gaining insight into physiological and pathological processes because pH values are related to various cell behaviors, such as cell activation, growth, proliferation, and apoptosis, as well as enzyme activity and protein function.^1^ In addition, individual organelles have their own regular pH range; the typical pH distribution of mammalian cells can vary from 4.4 to 6.5 for acidic lysosomes/endosomes to neutral (6.8 to 7.4) for cytoplasms/nuclei and 8.0 for basic mitochondria.^2^ Therefore, the detection of an aberrant pH in subcellular compartments, which is often associated with cell dysfunction, can be a promising diagnostic technique for many diseases, such as tumors, inflammation and Alzheimer’s disease.^3,4^ Fluorescence-based techniques have been proven to be the most effective way to monitor real-time intracellular and extracellular pH variation with high sensitivity and excellent read-out.^5^

Many fluorescent pH probes have been developed (as shown in recent reviews of the literature^1,5–13^), and generally, these sensors fall into the following groups: small organic dyes, dye-loaded nanoparticles (NPs; including those made from amphiphilic block co-polymers,^14–17^ silica^18,19^ and metal–organic-frames^20,21^), semiconducting small-molecular NPs,^2,22^ fluorescent proteins,^23,24^ water-soluble conjugated polymers,^25^ quantum dots (Qdots)^26,27^ and carbon dots (Cdots).^28–30^ These fluorescent probes have been demonstrated in a broad range of applications, including the determination of the pH values of whole cells or specific subcellular organelles, such as lysosomes,^31–34^ endosomes,^35,36^ cytosols^37,38^ and mitochondria.^39,40^ For example, a multi-compartment polymer nanostructure was used to achieve ratiometric pH sensing,^17^ and real-time cellular trafficking processes were monitored using a near-infrared (NIR) small-molecule pH indicator with fluorescence off/on features that were triggered by the lysosomes.^41^ Using a protease-activated ratiometric nanoprobe, the *in vivo* pH mapping of tumors was also demonstrated.^42^ Finally, using a block co-polymer design, a series of transistor-like pH responsive nanoprobes were obtained, and these ultra-pH-sensitive sensors were employed to quantify the pH of different endocytic organelles in live cells, to image endocytic trafficking and endosome maturation processes, and to study *in vivo* tumor microenvironments.^43,44^

Despite these impressive advances in fluorescence pH sensing, each of these groups of pH sensors have their limitations.^30,45^ Developing a photostable, ultra-bright and biocompatible fluorescence-based pH probe is still very much needed. In particular, our lab have been interested in carrying out single-molecule and single-particle pH sensing within the smallest spatially confined sub-cellular spaces, where pH changes are often large. However, to monitor such changes, the probe must be extremely photostable and bright. We also have a long-standing interest in studying the pH changes in individual synaptic vesicles,^46–49^ in which a single free proton would result in one pH unit change within a 40 nm vesicle. Existing pH probes cannot readily meet the requirements of this experiment, and we are thus motivated to develop a nanoparticle-based probe to satisfy the needs of such applications. We focus on NPs because it is possible to introduce a single 10–25 nm diameter NP into a synaptic vesicle by exploiting the exocytosis–endocytosis process^47,48^

In our design of a suitable fluorescent pH probe, we have made the following considerations: (1) brightness and pH sensitivity: to follow the fast pH changes at the level of a single probe, such as a single pH sensor confined within a 40 nm diameter synaptic vesicle, the probe must be very bright. At this single-molecule level, the pH sensitivity can depend more on the brightness of the probe than on the signal difference between the different pH values; (2) photostability: to monitor the pH change continuously from the same single probe over time with the high excitation power necessary for single-molecule sensitivity, the pH sensor must be highly photostable; (3) dynamic range: because pH changes tend to be very large within an ultra-small volume, it can be more important to design a single pH probe that is capable of achieving a wide dynamic range, rather than a pH probe with a sharp pH response; and (4) spectral real estate: the identification of a sub-cellular organelle, which is below the optical diffraction limit, often requires the use of multiple fluorescent markers, and it is desirable to reserve most of the visible wavelength range for use by other probes.

Semiconducting polymer dots (Pdots) recently have emerged as a new class of ultra-bright fluorescent nanoparticles.^50–58^ Their high brightness^53^ is of particular relevance. For example, a 20 nm diameter Pdot can have a molar extinction coefficient as high as 10^9^ M^–1^ cm^–1^, which is 2–3 orders of magnitude higher than that of a single small-molecule dye.^59^ Indeed, two pH sensitive Pdots were previously reported,^60,61^ but they were based on conjugation with a pH sensitive dye, fluorescein, and thus do not possess the high photostability needed for our applications.

We had previously developed a highly efficient NIR Pdot based on the incorporation of squaraine (Sq) derivatives into the backbone of the semiconducting polymer.^62^ According to the reported pH response of some Sq dyes,^26,63,64^ we thought these Pdots might be pH-responsive. However, upon testing, we found that these Pdots had almost no pH sensitivity. We hypothesized that this lack of pH response may be caused by the low concentration of Sq monomers present in the polymer. When we prepared and tested Pdots with a higher Sq content, we indeed found better pH sensitivity, but at the cost of a reduced quantum yield due to self-quenching of the Sq dyes.^63^

To solve the issue of self-quenching, this paper describes a series of copolymers with NIR Sq-derivative emitters, which were dendronized using different numbers of carbazoles as the bulky steric group. These dendronized Sq Pdots were pH sensitive and exhibited several useful features: (1) the self-quenching was minimized even when the incorporated molar ratio of the emitter was increased 5 fold (1% to 5%); (2) a higher incorporated molar ratio of Sq results in increased pH sensitivity; (3) an improved brightness and photostability of the NIR fluorescence; (4) ratiometric pH-sensing was made possible through blue emission from the polyfluorenes (not pH sensitive) and the NIR emission from the Sq (pH sensitive); (5) a wide dynamic range in pH sensing with a fast and reversible response; (6) chemically incorporating the pH probe into the backbone of the hydrophobic conjugated polymers prevents the possibility of dye leakage from the Pdots; and (7) the grafted bulky carbazole dendron pendant showed little influence on the narrow NIR emission of the Sq chromophores and the size of the Pdots.

While this single-Pdot pH sensor does not have a sharp pH response, it has very high single-particle brightness and photostability, a wide dynamic range, and optimal spectral characteristics. For applications limited by the photon budget, and especially for monitoring pH changes in the smallest confined sub-cellular spaces at the single-molecule level, this Pdot pH probe should highly complement currently available pH sensors.

## Results and discussion

We previously developed a series of Sq-based NIR polymers by incorporating the Sq dyes into the backbone of a poly(9,9-dioctylfluorene) (PFO) host.^62^ Using these polymers, we created highly efficient NIR Pdots with a large Stokes shift arising from efficient through-bond energy transfer.^65^ To minimize the self-quenching of the Sq emitter, the effective Sq feeding ratio was limited to be around 1.5 mol%. If the feeding ratio of the Sq monomer was increased to 3.0 mol% during polymerization, the absolute fluorescence quantum yield (*Φ*_f_) of the Pdots dramatically decreased from 0.30 to 0.08, which could be ascribed to the severe concentration quenching of the Sq dye. The low effective doping ratio (∼1.5 mol%) of the Sq emitter impeded the Pdots’ sensing property, mainly because the final concentration of the pH probe (Sq emitter) in the tested PBS buffer was too low.

**Scheme 1:**
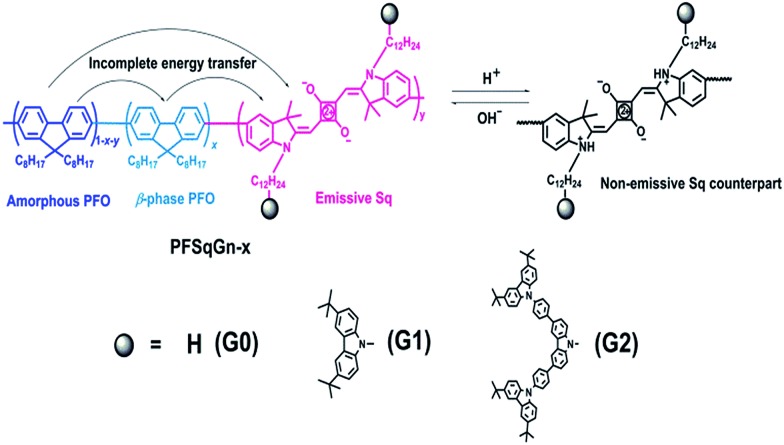
Chemical structure of the PFSqG*n* polymers and the mechanism for the ratiometric Pdots-based pH sensor.

Bulky steric groups,^66^ especially dendrimers,^67^ can suppress fluorescence concentration quenching in the condensed state. Therefore, we designed and synthesized two Sq-based monomers (SqG*n*) grafted with different generations (G0, G1, G2) of the carbazole dendron pendant. They were incorporated into the backbone of the PFO to obtain the PFSqG*n* polymers (Scheme 1). The PFSqG0 polymer without carbazole derivatives was synthesized as a reference, which had almost the same structure as the polymer/Pdot in our previous work except for the different alkyl chain lengths of the Sq dye. The detailed synthetic routes and characterizations of the PFSqG*n* polymers are in the ESI.†

After the semiconducting polymers with different Sq generations (SqG*n*) and different feeding molar ratios were synthesized and characterized, the corresponding Pdots were prepared using the well-established nanoprecipitation method.^68^ To introduce functional groups for bioconjugation, we used polystyrene with carboxylated polyethylene glycol (PS-PEG-COOH) as described in our prior publications;^69^ here, we found the optimal weight content of PS-PEG-COOH in the Pdots was 20% (Fig. S1†).

**Fig. 1:**
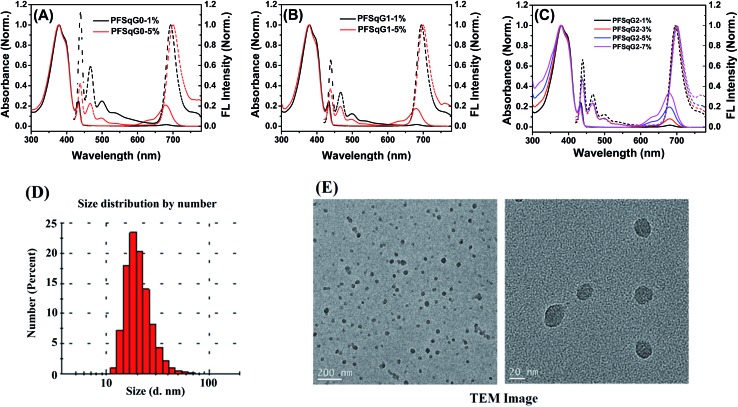
Normalized absorption and FL spectra of the PFSqG*n-x* Pdots in DI water (A–C); a DLS size distribution histogram (D) and high-resolution TEM images of the PFSqG2-5% Pdots (E).

Fig. 1A–C shows the absorption and fluorescence (FL) spectra of the PFSqG*n* Pdots (0.01 g L^–1^ in DI water). These Pdots exhibited three absorption peaks at around 380, 432, and 680 nm, which can be ascribed to the absorption of the amorphous PFO backbone, β-phase PFO,^70^ and Sq monomer, respectively. There were only two emission peaks in the FL spectra from the β-phase PFO and the Sq monomer, which was due to efficient energy transfer from the amorphous PFO to the β-phase PFO and Sq dye. Both the relative absorption and FL intensity in the NIR region were enhanced with higher molar ratios of the emitter. Here, emission from the β-phase PFO was used as the internal reference to obtain a ratiometric pH sensor. Without this β-phase emission, ratiometric pH sensing could not be achieved because nearly complete energy transfer from the amorphous PFO to the NIR squaraine dye would occur (Fig. S2†).

As summarized in Table 1, all of the PFSqG*n*-1% Pdots showed comparable *Φ*_f, NIR_ values (the acquired *Φ*_f_ value of the NIR region from 650–780 nm) of around 21% because the quenching effect was negligible at such a low molar ratio of Sq monomers. However, when the molar ratio of the Sq monomers was increased to 5%, the *Φ*_f, NIR_ value of PFSqG0-5% Pdots rapidly decreased to 6.1%, while the *Φ*_f, NIR_ value of PFSqG2-5% Pdots remained as high as 11.7%. These data show that the self-quenching of the Sq monomers was reduced by the introduction of bulky steric carbazole dendrons. Because the carbazole dendron was grafted to the Sq using a long alkyl chain spacer, it did not impose any disadvantages on the shape of the NIR emission spectra. The Pdots with carbazole dendrons on the Sq emitters did not change their peak emission wavelength or bandwidth. Maintaining the narrow bandwidth is especially important for Sq Pdots as they were designed for multiplexed biological imaging and analyses. Additionally, the Pdots with carbazole dendrons showed comparable sizes to regular Pdots without the dendrons, and they had an average particle diameter of 23 nm, as measured using dynamic light scattering (DLS) (Table 1). Therefore, the carbazole dendron pendants did not affect the inner packing conditions of these PFSqG*n*-5% Pdots.

**Table 1:** Photophysical properties and DLS size of the PFSqG*n* Pdots

Pdots	*λ*_abs_ (nm)	*λ*_FL_ (nm)	*Φ*_f, NIR_ (%)	Size (nm)
PFSqG0-1%	379/432/680	439/693	21.2	22.8
PFSqG0-5%	378/432/680	438/700	6.1	23.3
PFSqG1-1%	378/433/681	439/695	22.4	23.2
PFSqG1-5%	379/432/680	438/699	7.6	23.0
PFSqG2-1%	380/434/681	439/696	20.8	23.9
PFSqG2-3%	380/433/680	439/698	15.8	22.1
PFSqG2-5%	379/433/680	438/701	11.7	22.7
PFSqG2-7%	379/433/679	438/702	7.8	24.1

**Fig. 2:**
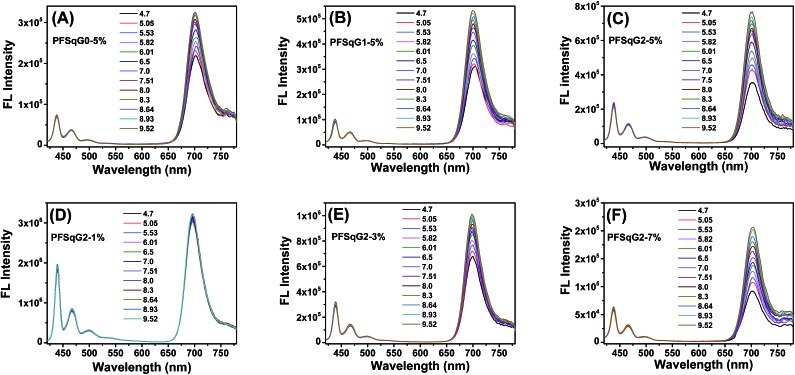
The emission spectra of the PFSqG*n*-5% (A–C) and PFSqG2-*x* (D–F) Pdots in PBS buffer with different pH values.

Next, we studied the pH-responsive behavior of these Pdots in PBS buffer with different pH values. From the pH-dependent FL spectra (Fig. 2), we found that the FL intensity at 438 nm originating from the β-phase PFO segment was almost constant at different pH values; the FL intensity at around 700 nm originating from the SqG*n* emitters changed as the pH values varied. This result suggested that we could use these Pdots as ratiometric pH sensors, relying on the blue emission as an internal reference.

**Fig. 3:**
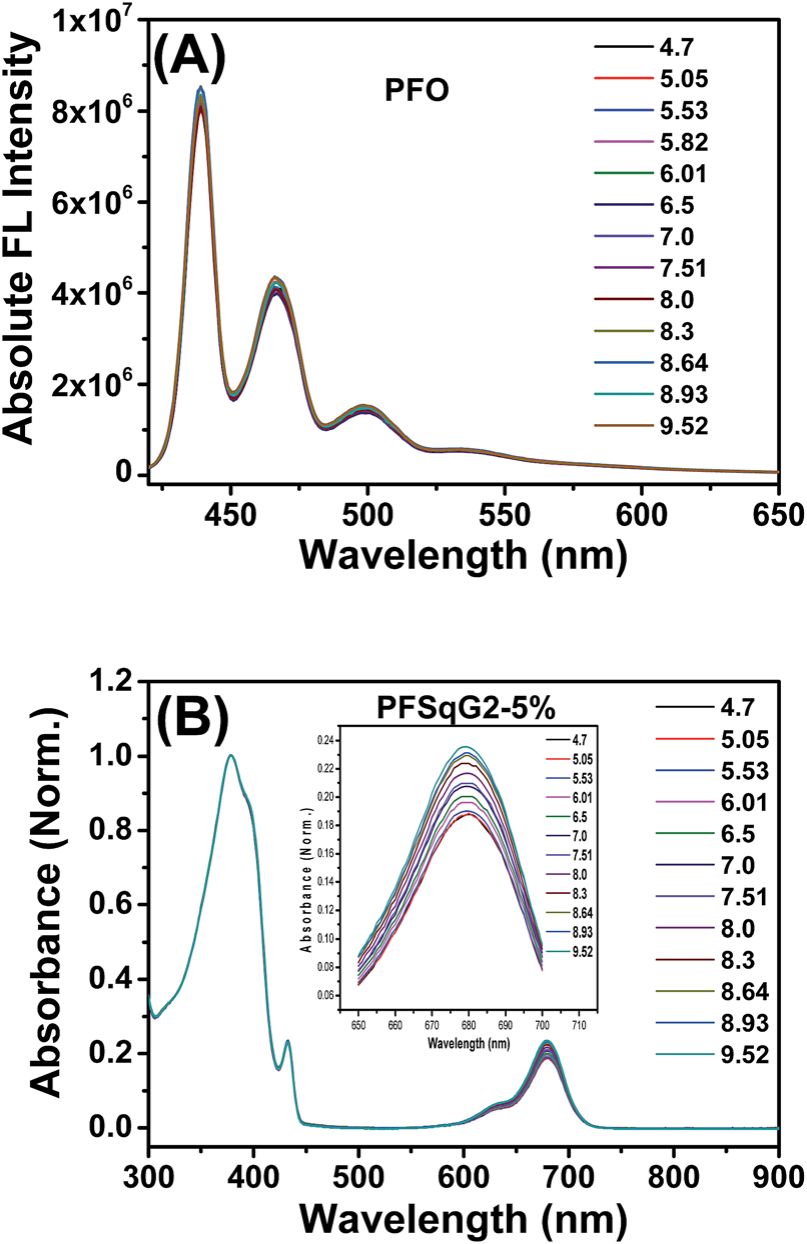
Absolute fluorescence (FL) spectra of the PFO Pdots (A) and the normalized absorption spectra of the PFSqG2-5% Pdots (B) with different pH values in PBS buffer.

To better understand the mechanism of the ratiometric pH response of the Pdots, we first used the same protocol to prepare the bare PFO Pdots and checked their pH sensitivity. As shown in Fig. 3A, the FL spectra of the PFO Pdots show that they are almost pH-independent, suggesting that the pH sensitivity of the PFSqG*n* Pdots came from the SqG*n* monomers. This hypothesis was verified using the absorption spectra of the PFSqG*n* Pdots. Fig. 3B shows the normalized absorption spectra of the PFSqG2-5% Pdots in PBS buffer with different pH values. The relative absorbance intensity of the Sq monomer at around 680 nm continuously rose with increasing pH value. Therefore, we believe there is an equilibrium between the emissive and non-emissive protonic counterparts of the Sq monomers (Scheme 1). When the aqueous medium is acidified, more indoline segments of the Sq monomers will be protonated so that they lose their electron-donating ability to the central squaryl ring as the electron acceptor, and so its intramolecular charge transfer (ICT) absorption becomes weaker. Conversely, its ICT absorption becomes stronger in basic aqueous environments due to the deprotonation of the indoline segments. It is possible that protonation could occur on the oxygen atom of the squaraine unit rather than on the nitrogen atom of the indoline. However, based on the work of Ros-Lis *et al.*, protonation on the oxygen atom would result in a slightly red shifted and/or greatly blue-shifted absorption band accompanied by a reduction in the intensity of the normal absorption band.^71^ We checked the absorption spectra of our G0Sq monomer in acidic solutions, and found only the main peak at around 640 nm decreased in intensity, and no new absorption band was found (Fig. S3†), thus indicating that the protonation was on the nitrogen atom.

**Fig. 4:**
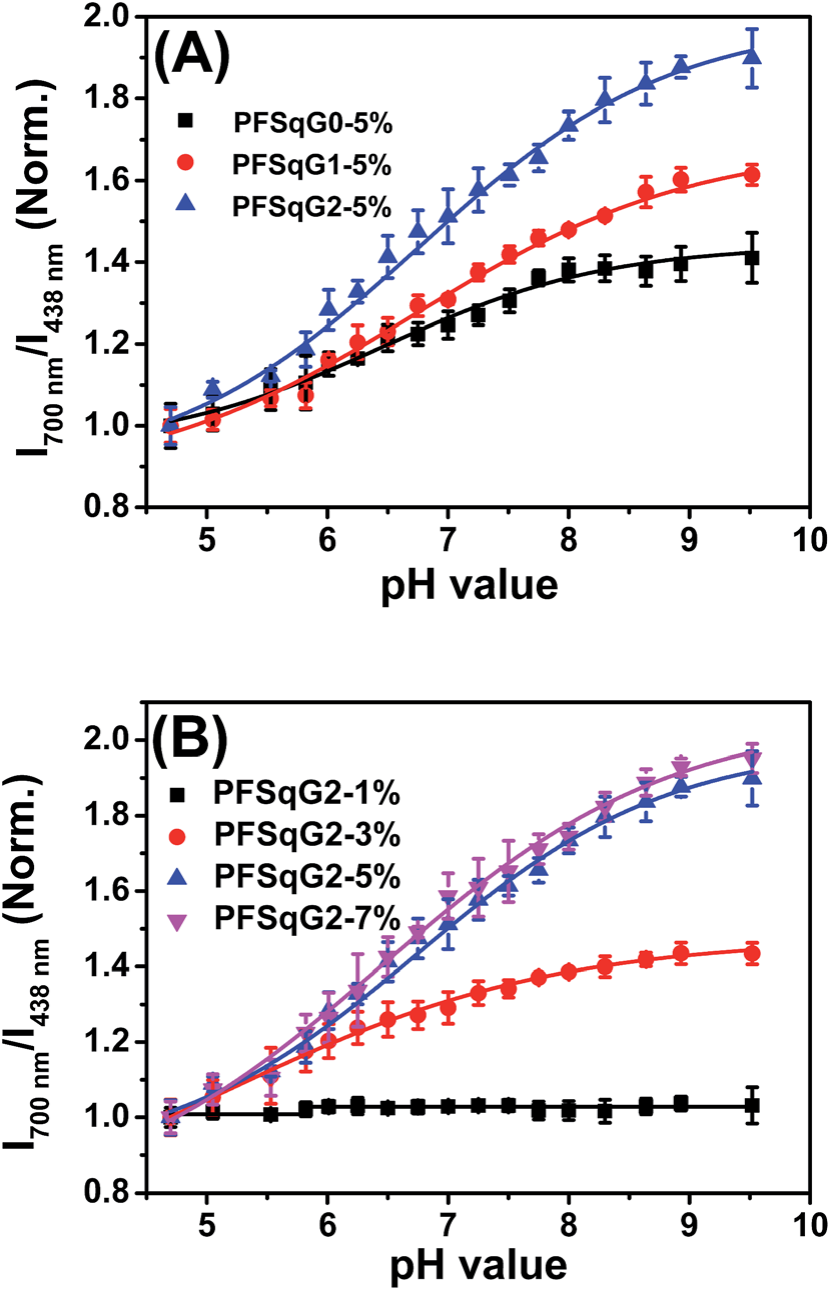
Normalized plot of *I*_700 nm_/*I*_438 nm_ *vs.* pH value for the PFSqG*n*-5% (A) and PFSqG2-*x* (B) Pdots.

Among the Pdots tested, the PFSqG*n*-1% Pdots showed very poor pH sensitivity (see PFSqG2-1% in Fig. 2D as an example) even though they possess the highest *Φ*_f, NIR_ value. This was caused mainly by the low content of Sq monomers in the Pdots. For instance, the SqG2 monomer concentration of the PFSqG*n*-1% Pdots in PBS buffer solution was estimated to be only 2.4 × 10^–7^ mol L^–1^. The other reason is that the strong blue reference emission resulted in a lower FL intensity ratio (*I*_700 nm_/*I*_438 nm_). As shown in Fig. 2A–C, the higher Sq dye content of three PFSqG*n*-5% Pdots resulted in much better pH sensitivity. It is evident that the PFSqG2-5% Pdots show the best pH sensitivity from their pH-responsive dynamic curves (Fig. 4A). This was because of the improved *Φ*_f, NIR_ values of the PFSqG2-5% Pdots that made a more distinct change in the FL intensity as a function of pH. In addition, the decreased fluorescence self-quenching by the introduction of the carbazole dendron also enabled the PFSqG2-5% Pdots to be twice as bright as the PFSqG0-5% Pdots. Besides demonstrating the importance of introducing bulky groups of Sq monomers to get brighter Pdots with better pH sensitivity, we also tried to optimize the feeding ratio of the SqG2 monomers. Polymers with different feeding molar ratios of 1%, 3%, 5% and 7% were synthesized, and the pH sensitivities of the corresponding Pdots were compared. From the FL spectra (Fig. 2C–F) and the pH-responsive curves (Fig. 4B), we see there is greater pH sensitivity with the higher feeding ratios of the SqG2 monomers. Compared to the PFSqG2-5% Pdots, the PFSqG2-7% Pdots showed slightly improved pH sensitivity, but relatively low NIR-emission brightness. Considering the trade-off between pH sensitivity and brightness, we used the PFSqG2-5% Pdots for the additional studies.

As shown in Scheme 1, there is energy transfer from the amorphous PFO to the β-phase of the PFO and Sq dye as well as possible energy transfer from the β-phase of the PFO to the Sq dye. This results in two emission bands from the blue β-phase PFO and NIR Sq dye. The stronger absorption of the Sq dye leads to weaker blue emission, but not correspondingly higher NIR emission because of the trade-offs between more energy transfer and concentration quenching of the Sq dye. In fact, both the absolute-blue and NIR-emission intensities gradually reduced when the Sq content increased from 1% to 3%, 5% and 7% (Fig. S4†). In this case, how do we explain the Pdots that show stronger NIR emission combined with enhanced NIR absorption under higher pH conditions? A reasonable explanation is that the presence of the fluorescence quencher was effectively suppressed under high pH conditions. The quencher might be the non-emissive Sq monomer counterpart or a Sq dimer created *via* π–π stacking and intermolecular H-band interactions.^72^ In principle, these two species can be reduced *via* the deprotonation of the indolium unit under basic conditions.

**Fig. 5:**
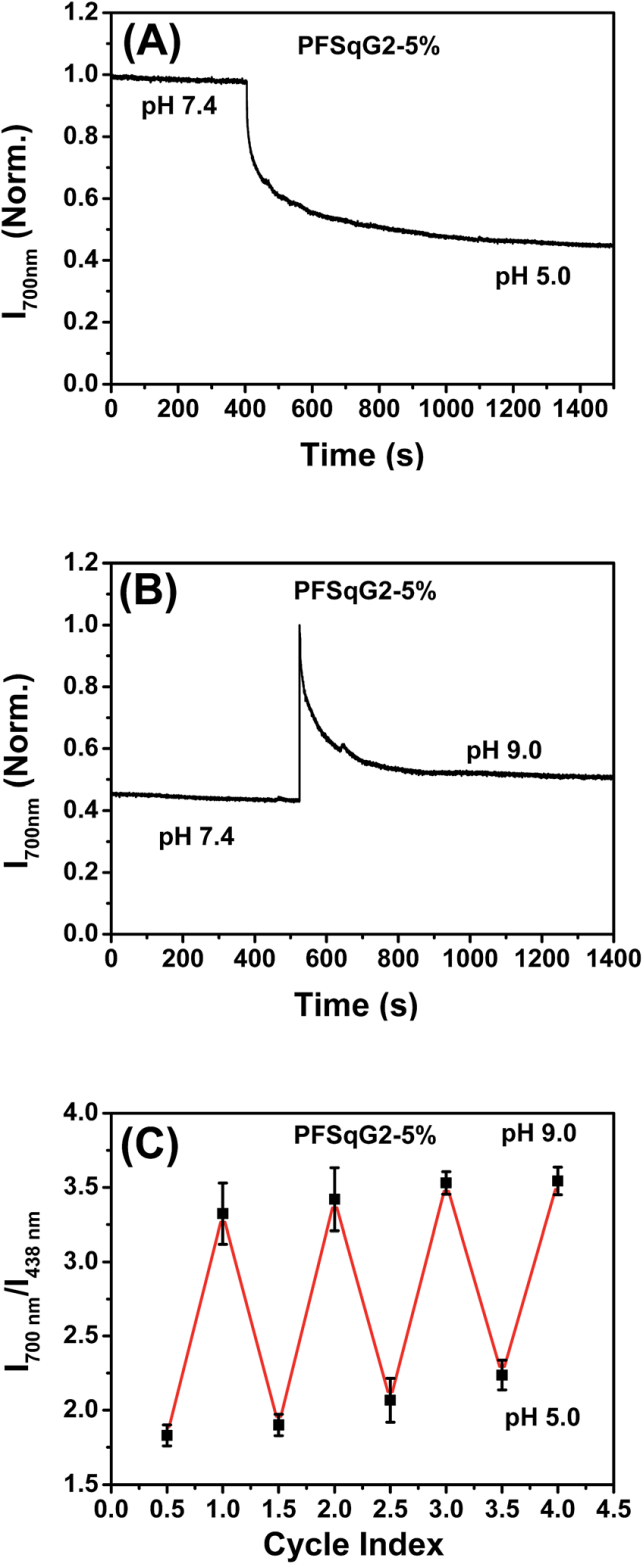
The rapid (A and B) and reversible (C) pH response of the PFSqG2-5% Pdots.

In addition to the wide pH-sensing dynamic range from 4.7 to 9.5, which is partly derived from the near-neutral p*K*_a_ value of the Sq-based probe (calculated to be 6.85), the PFSqG2-5% Pdots also showed excellent pH response rates under both acidic and basic conditions. We prepared the PFSqG2-5% in neutral PBS buffer (pH 7.4) and then tuned the pH value to 5.0 or 9.0 using 1 mol L^–1^ HNO_3_ or NaOH. Their time-dependent FL intensities at 700 nm were recorded, as shown in Fig. 5A and B. When the pH changed from neutral to acidic, the Pdots needed about 16 minutes to achieve a saturated pH response. When the pH was tuned from neutral to basic, the Pdots gave an over-saturated pH response and then achieved their final equilibrium in around 6 minutes. The results also were confirmed using the corresponding time-dependent FL spectra (Fig. S5†). In these experiments, the actual pH response of the Pdots is likely to be faster, and is masked by the time required for the mass transfer in these bulk experiments. The pH sensor based on the Pdots demonstrated good reversibility between the acidic (pH 5.0) and basic (pH 9.0) media, as shown in Fig. 5C.

**Fig. 6:**
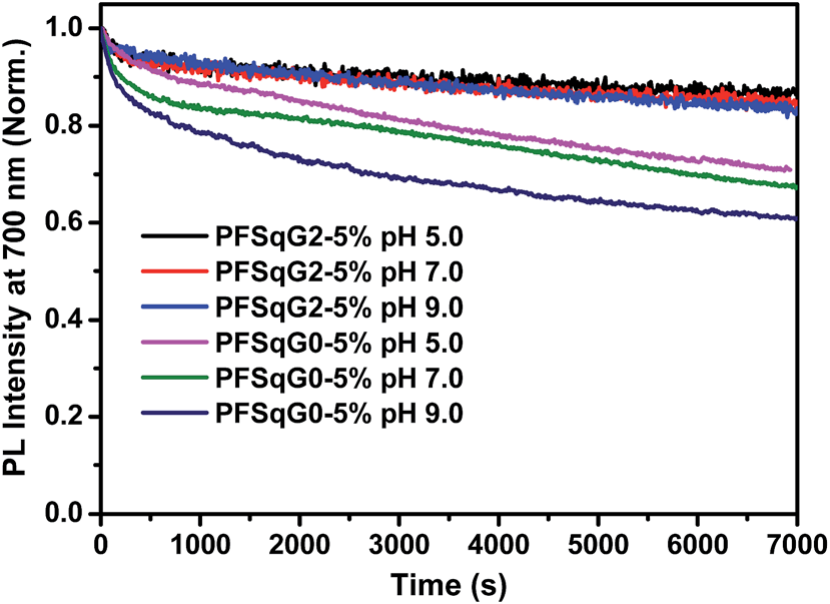
Photostability of the PFSqG0-5% and PFSqG2-5% Pdots at different pH values.

The photostability of the fluorescent indicator is always a challenging and critical issue, especially during time-lapse fluorescence imaging experiments or in single-molecule studies. We have shown previously that most kinds of Pdot exhibit much better photostability than small organic dyes, and have comparable photostabilities to those of inorganic Qdots^50,73,74^ Here, we found that the photostability of the Pdots was further improved after grafting with the electron-donating carbazole dendron. We compared the photostability of the PFSqG0-5% and PFSqG2-5% Pdots at pH 5.0, 7.0 and 9.0. Fig. 6 illustrates the time-dependent FL intensities of the PFSqG0-5% and PFSqG2-5% Pdots at different pH values after being irradiated for 7000 s. The PFSqG2-5% Pdots could remain at 86.3%, 84.9% and 83.6% of the initial FL intensity at pH values of 5.0, 7.0 and 9.0, respectively, but the corresponding values of the PFSqG0-5% Pdots were only 70.9%, 67.3% and 60.9%. We believe that the grafted carbazole derivatives improved the photostability of the PFSqG2-5% Pdots.

**Scheme 2:**
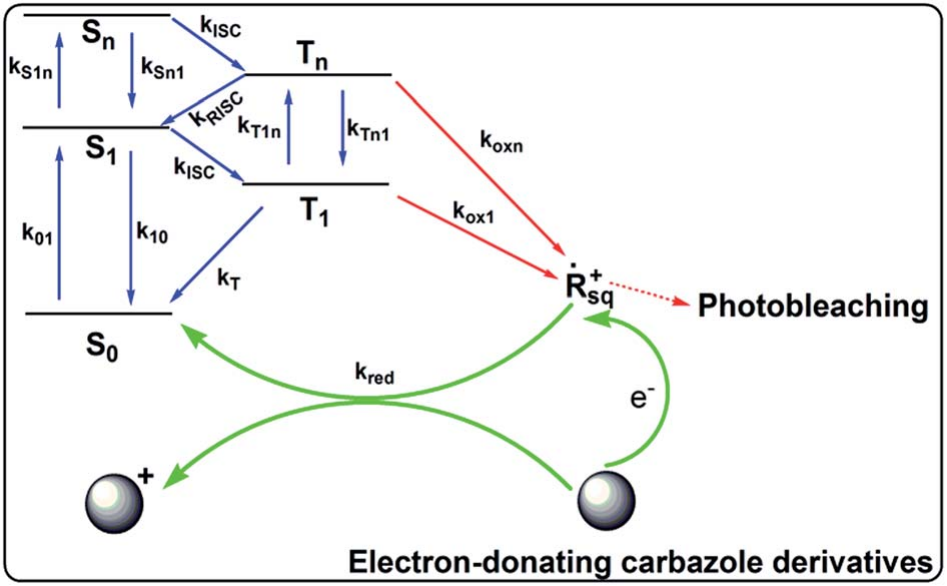
The mechanism for the improved photostability of the PFSqG2-5% Pdots.

Scheme 2 illustrates the hypothesized mechanism for the improved photostability of the PFSqG2-5% Pdots. After the dye is excited, a number of the singlet excitons are transferred to the triplet state through intersystem crossing. The triplet excitons are relatively long-lived compared to the singlet ones, thus allowing them to be more easily oxidized by the reactive oxygen species (ROS) in the environment to form an electron-deficient radical intermediate. Then, the radical may be converted to a non-emissive product due to irreversible covalent modifications. However, if there is an electron donor, such as the carbazole unit on the Sq as in our work, the radical can withdraw an electron from the donor and be reduced to the ground state. This reduction stops photobleaching.^75^ To verify our hypothesis, we checked the stability of the SqG0, SqG1 and SqG2 monomers in tetrahydrofuran (THF) solution because it is well known that the THF solvent can easily form some peroxide that can facilitate the photochemical damage of the Sq dye. The results (Fig. S6†) show that the SqG0 monomer in THF was almost completely degraded within 24 hours of being under the ambient light conditions; in contrast, the absorbance of the SqG1 and SqG2 monomer dyes at around 650 nm showed negligible variation. Therefore, it appears that the electron-donating carbazole derivatives indeed suppressed the photobleaching of the SqG2 monomers inside the Pdots.

**Fig. 7:**
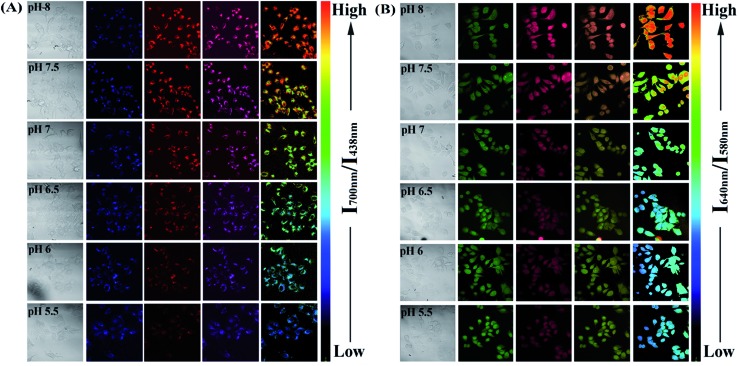
Intracellular pH mapping using the PFSqG2-5% Pdots (A) and SNARF-1 (B). The Pdots-loaded cells were incubated at 37 °C for 15 min in buffers with various pH values in the presence of 10 μM nigericin. The channels, from left to right, are the bright field, blue channel from PFO emission, red channel from NIR emission, merged and ratio channel.

Since the PFSqG2-5% Pdots appeared to be a promising pH sensor, especially in the pH range 5.0–9.0, we investigated the feasibility of using them for ratiometric fluorescence imaging within the intracellular environment of living cells. We first demonstrated that the PFSqG2-5% Pdots had good biocompatibility, as shown by 3-(4,5-dimethylthiazol-2-yl)-2,5-diphenyltetrazolium bromide (MTT) assays (Fig. S7†). We felt that establishing the biocompatibility of the PFSqG*n* Pdots was important before moving ahead with further biomedical applications. An intracellular calibration experiment was first carried out in MCF-7 cells with the H^+^/K^+^ ionophore nigericin to quantify the intracellular pH value. The uptake of the Pdots was achieved by exposing the cells to the PFSqG2-5% Pdots for 24 hours. After washing off the extracellular Pdots, the Pdots-loaded MCF-7 cells were microscopically inspected. Confocal fluorescence images were recorded using excitation at 405 nm and by collecting the fluorescence emission in two separate channels. As illustrated in Fig. 7A, the FL intensity of the NIR-channel (red pseudocolor) increased with pH value, whereas the internal-control channel (blue pseudocolor) showed negligible change in the FL intensity. The discrimination between the acidic and alkaline intracellular environments was obvious from the merged channel, with the merged color changing from purple to pink. The color ratio in Fig. 7A was used to calibrate the intracellular pH, where the intensity ratio of the two emission colors (NIR and blue) was calculated pixel-to-pixel and then pseudocolored. The results show an obvious change from pH 5.5 to 8.0, which was consistent with the results acquired from the pH-dependent FL spectra.

To verify the validity of the PFSqG2-5% Pdots for pH sensing, a commercially available pH probe, SNARF-1, was also applied to determine the intracellular pH values of the MCF-7 cells. SNARF-1 displayed a different fluorescence performance in different pH values compared to the PFSqG2-5% Pdots. SNARF-1 exhibited an isosbestic point at 600 nm and a spectral shift of the maximum emission (Fig. S8A†). Although the results obtained from SNARF-1 (Fig. 7B) matched with those from using the PFSqG2-5% Pdots in the pH range 6.5 to 8.0, it was hard to use SNARF-1 for intracellular pH profiling within the whole physiological pH range, especially towards the acidic side of the range (Fig. 7B and S8B†).

**Fig. 8:**
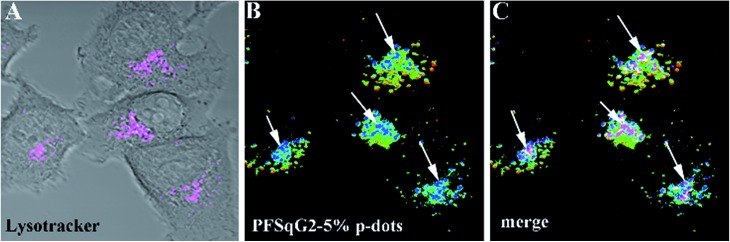
Intracellular cell mapping using the PFSqG2-5% Pdots. A differential interference contrast (DIC) image composite using the LysoTracker channel (A); ratiometric images of MCF-7 cells incubated with the PFSqG2-5% Pdots (B); and composite images of the LysoTracker channel and the ratiometric images (C).

Encouraged by the successful results, we next applied the Pdots for intracellular pH mapping. As depicted in Fig. 8, the Pdots were unevenly distributed inside the cytoplasm but not the nucleoplasm. To elucidate the nature of the color variation displayed in the ratiometric images highlighted by the Pdots (Fig. 8B), LysoTracker Green DND-26, a selective probe for acidic compartments in live cells, was co-incubated with the Pdots. To discriminate the signal of the LysoTracker from those of the Pdots’ ratiometric pseudocolor, we painted the LysoTracker channel with a pink pseudocolor, as shown in Fig. 8A. To further clarify the localization of the LysoTracker and Pdots, differential interference contrast (DIC) images of the MCF-7 cells were also collected simultaneously with the fluorescence images (Fig. 8C). The co-localization of the LysoTracker with the Pdots clearly demonstrated that the ratiometric pH-sensing Pdots had mainly accumulated in the acidic organelles within the intracellular environment, such as the lysosomes, which have a lower pH value than the surrounding cytoplasms (Fig. 8C).

**Fig. 9:**
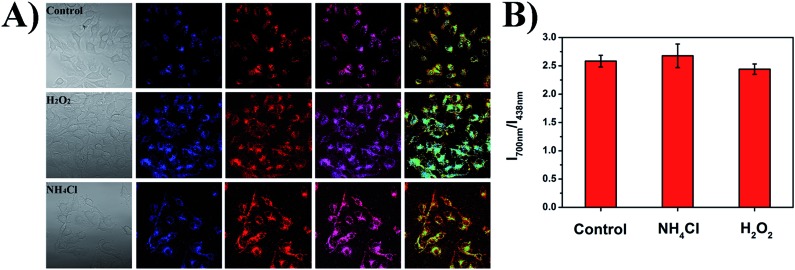
The impact of NH_4_Cl and H_2_O_2_ on intracellular pH measured using the PFSqG2-5% Pdots. The intracellular pH of the cells treated with NH_4_Cl and H_2_O_2_ in pH 7.4 PBS buffer solution (A), and the calculated ratio of *I*_700 nm_/*I*_438 nm_ of the PFSqG2-5% Pdots for the control and treated cells (B).

As shown in Fig. 9, the small intracellular pH fluctuations caused by different chemical substances were also studied to show the robustness of the ratiometric pH measurements of the PFSqG2-5% Pdots. H_2_O_2_ and NH_4_Cl can acidify and basify live cells to a small extent (0.1–0.2 pH unit), respectively.^76,77^ The comparative study with SNARF-1 (Fig. S9†) proved that the PFSqG2-5% Pdots effectively detected the tiny intracellular pH changes of the live MCF7 cells.

## Conclusions

By introducing a large steric carbazole dendron to the side chain of the NIR-emission squaraine dye, we obtained a series of NIR-emitting Pdots with simultaneously suppressed self-quenching and photobleaching. The improved *Φ*_f, NIR_ values of the Pdots with high squaraine dye content led to superior pH sensing performance. By controlling the energy transfer from the amorphous PFO to the β-phase PFO and Sq dye, we created ratiometric pH-sensitive Pdots that have both blue and NIR emission. The Pdots showed rapid and reversible responses within the whole physiological pH range. Intracellular pH mapping was successfully performed in MCF-7 cells using PFSqG2-5% Pdots. Within the pH range 6.5 to 8.0, the PFSqG2-5% Pdots showed comparable pH sensitivity to a commercial organic-dye based pH probe, SNARF-1, and in acidic surroundings (4.7–6.5), the PFSqG2-5% Pdots had better performance than SNARF-1. Based on these results, we anticipate our Pdots to be promising candidates for monitoring intracellular pH values in particular, and pH sensing in general.

## Conflicts of interest

The authors declare no competing financial interest.
