# Supplementary material for: Highly photostable wide-dynamic-range pH sensitive semiconducting polymer dots enabled by dendronizing the near-IR emitters
Source: Chem Sci. 2017 Sep 4;8(10):7236–45. doi: 10.1039/c7sc03448b (PMC5633788; doi:10.1039/c7sc03448b)
Supplement: Supplementary file 1 [file SC-008-C7SC03448B-s001.pdf]

Electronic Supplementary Material (ESI) for Chemical Science.
This journal is © The Royal Society of Chemistry 2017

SUPPORTING INFORMATION

Highly Photostable Wide-Dynamic-Range pH Sensitive Semiconducting Polymer Dot Enabled by Dendronizing the Near-IR Emitters

Lei Chen, Li Wu, Jiangbo Yu, Chun-Ting Kuo, Tengyue Jian, I-Che Wu, Yu Rong and Daniel T. Chiu*

Department of Chemistry, University of Washington, Seattle, Washington 98195, United States

Experimental Section

1. Synthesis of PFSqGn-*x* polymers

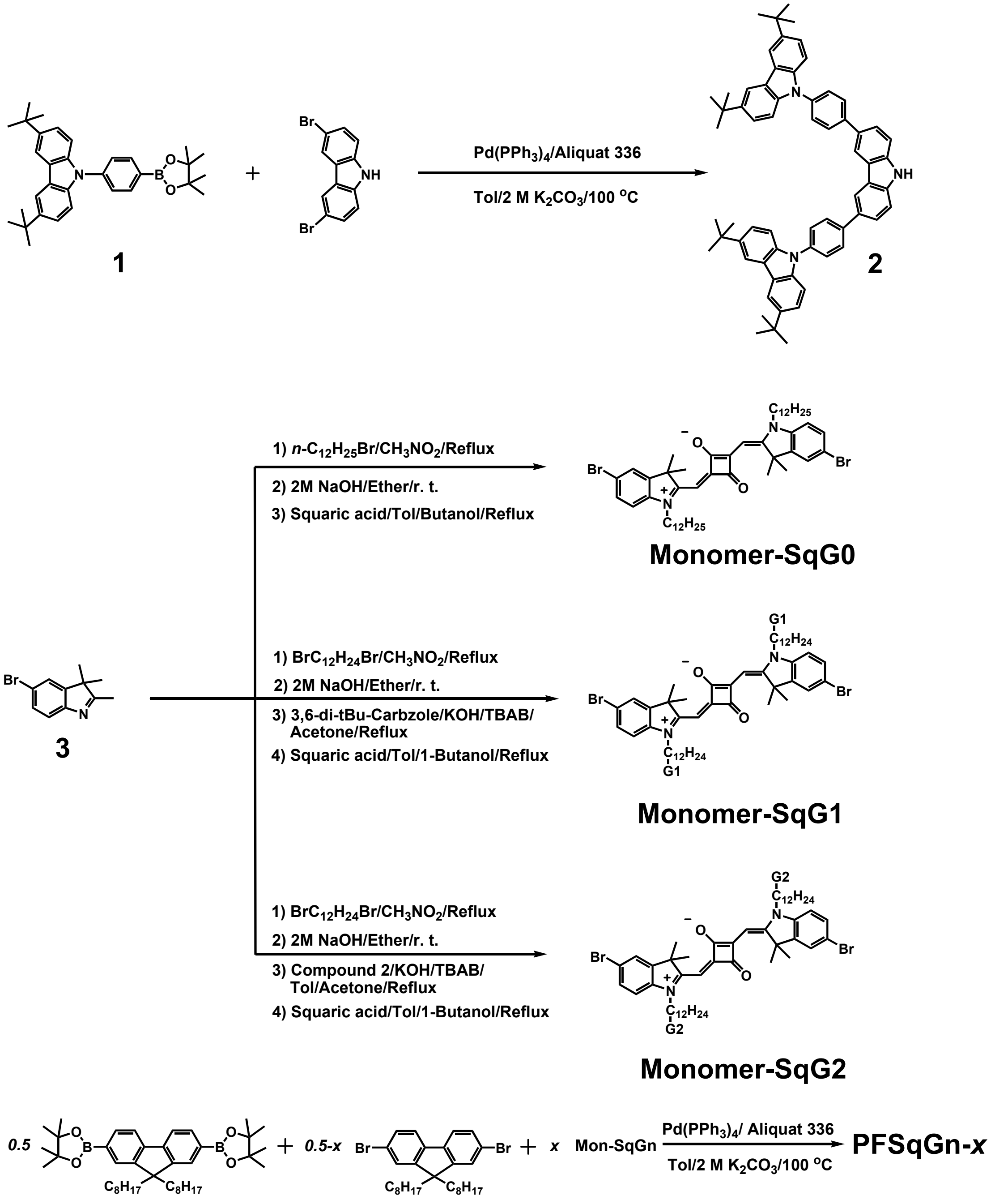

Scheme S1. The synthetic routes of squaraine-based monomers and polymers.

3,6-di-tert-butyl-carbazole, 3,6-dibromo-carbazole and squaric acid were purchased from TCI America and used directly. 9,9-dioctylfluorene-2,7-diboronic acid pinacol ester and 2,7-dibromo-9,9-dioctylfluorene monomers were purchased from Sigma-Aldrich and recrystallized twice in ethanol for polymerizations. 3,6-di-tert-butyl-9-(4-(4,4,5,5-tetramethyl-1,3,2-dioxaborolan-2-yl)phenyl)-9H-carbazole (**1**)[1] and 5-Bromo-2,3,3-trimethylindolenine (**3**) were synthesized using the same process from literature.[2]

Synthesis of compound 2

A mixture of compound **1** (3.5 g, 7.2 mmol), 3,6-dibromo-carbazole (980 mg, 3.0 mmol), $Pd(PPh_3)_4$ (100 mg), Aliquat 336 (3 drops), toluene (60 mL), 2 M K_2CO_3 (20 mL) was reacted at 100 ^oC under N_2 gas for overnight. After cooling, the mixture was washed with water three times and concentrated under vacuum to get raw product. The raw product was purified by column chromatography (Hexane: Dichloromethane (DCM), 4:1 as eluent) to get 2.10 g white solid with yield of 80.1%. ^1H NMR (500 MHz, $CDCl_3$, δ): 8.49 (s, 2H), 8.17 (s, 4H), 7.94 (d, J = 3 Hz, 4H), 7.81 (d, J = 3.4 Hz, 2H), 7.67 (d, J = 3 Hz, 4H), 7.59 (d, J = 3.4 Hz, 2H), 7.50 (d, J = 3.4 Hz, 4H), 7.45 (d, J = 3.4 Hz, 4H), 1.48 (s, 36H); ^{13}C NMR (75 MHz, $CDCl_3$, δ): 142.88, 140.66, 139.66, 136.80, 132.48, 128.45, 127.06, 125.65, 124.20, 123.61, 123.47, 118.97, 116.25, 111.18, 109.34, 34.75, 32.05; ESI-MS (m/z): 874.0 (M^+).

Synthesis of Monomer-SqG0

The monomer was obtained by a multistep synthesis, which is similar to a procedure we have reported previously.[3] Briefly, a mixture of compound **3** (500 mg, 2.10 mmol), 1-bromododecane (1.05 g, 4.2 mmol) and nitromethane (15 mL) was refluxed under N_2 gas for overnight. After cooling, the nitromethane solvent was removed under reduced pressure. Diethyl ether (50 mL) was added and filtered. The filter cake was washed with diethyl ether several times to remove most of the excess 1-bromododecane and other impurities. The resultant solid was vigorously stirred in mixed diethyl ether (20 mL) and 2 M NaOH (20 mL) for 1 h. The organic layer was separated and washed with DI water three times, then dried and evaporated to get 637 mg (1.6 mmol) yellow oil. It then was reacted with squaric acid (74 mg, 0.65 mmol) in mixed toluene and 1-Butanol solvent (15 mL: 15 mL) under N_2 gas. After refluxed for overnight, the solvent was evaporated under vacuum. The residual was further purified by column chromatography (DCM as eluent) to get 369 mg of dark green products. The total yield calculated from compound **3** starting material is 39.9%. ^1H NMR (500 MHz, $CDCl_3$, δ): 7.45 (s, 2H), 7.42 (d, J = 3.4 Hz, 2H), 6.84 (d, J = 3.4 Hz, 2H), 5.95 (s, 2H), 5.30 (s, 2H), 3.92 (Br, 4H), 1.78 (s, 16H), 1.39 ~ 1.25 (m, 40H), 0.88 (t, J = 2.4 Hz, 6H); ^{13}C NMR (75 MHz, $CDCl_3$, δ): 182.13, 180.37, 169.49, 144.26, 141.58, 130.68, 125.72, 116.59, 110.66, 87.12, 49.36, 43.89, 31.88, 29.48, 29.44, 29.29, 27.06, 22.65, 14.07; ESI-MS (m/z): 891.0 (M^+).

Synthesis of Monomer-SqG1

A mixture of compound **3** (952 mg, 4.0 mmol), 1, 12-dibromododecane (13.1 g, 40.0 mmol) and nitromethane (50 mL) was refluxed under N_2 gas overnight. After cooling to room temperature, the mixture was further cooled at 4 ^oC for 2 hours. Then it was filtered to remove most of the excess 1, 12-dibromododecane. The filter cake was washed with cooled nitromethane several times. Then the filtrate was combined and evaporated under reduced pressure. Diethyl ether (50 mL) was added to get gray precipitate, filtered again and washed with diethyl ether. The obtained solid was vigorously stirred in mixed diethyl ether (30 mL) and 2 M NaOH (30 mL) for 1 h. The ether layer was separated and washed with DI water three times, then dried and evaporated to get 1.2 g (2.5 mmol) yellow oil. After workup, 3,6-di-tert-butyl-carbazole (1.4 g, 5.0 mmol), potassium hydroxide (1.4 g, 25 mmol), Tetrabutylammonium bromide (**TBAB**, 160 mg, 0.5 mmol) and acetone (60 mL) was added and refluxed overnight under N_2 gas. After that, the acetone solvent was evaporated. The residual was dissolved by DCM and extracted with DI water three times. Then DCM solvent was evaporated to get brown oil, which was then reacted with squaric acid (114 mg, 1.0 mmol) in mixed toluene and n-Butanol solvent (30 mL: 30 mL) under N_2 gas. After being refluxed overnight, the solvent was evaporated under vacuum. The residual was further purified by column chromatography (DCM as eluent) to get 784 mg red-brown products. The total yield calculated from compound **3** starting material is 27.1%. ^1H NMR (500 MHz, $CDCl_3$, δ): 8.09 (s, 2H), 7.50 (d, J = 3.4 Hz, 4H), 7.44 (s, 2H), 7.40 (d, J = 3.4 Hz, 2H), 7.30 (d, J = 3.4 Hz, 4H), 6.82 (d, J = 3.4 Hz, 2H), 5.94 (s, 2H), 4.23 (t, J = 2.8 Hz, 4H), 3.91 (Br, 4H), 1.87 ~ 1.77 (m, 20H), 1.45 (s, 36H), 1.38-1.23 (m, 32H); ^{13}C NMR (125 MHz, $CDCl_3$, δ): 180.27, 169.44, 144.26, 141.37, 138.98, 130.71, 125.75, 123.21, 122.64, 116.62, 116.27, 110.70, 108.03, 87.10, 49.38, 43.90, 43.20, 34.68, 32.11, 29.54, 29.47, 29.36, 29.18, 27.38, 27.08, 27.00; ESI-MS (m/z): 1445.9 (M^+).

Synthesis of Monomer-SqG2

Monomer SqG2 was synthesized as the similar procedure as **Monomer-SqG1**, using compound **2** instead of 3,6-di-tert-butyl-carbazole. The only difference was in using a mixed solvent (Toluene: acetone, 2: 1) to improve the solubility of compound **2** during step 3. After the **Monomer-SqG2** residue was obtained, it was precipitated in methanol three times to remove some impurities first and then further purified by column chromatography (DCM as eluent) to obtain 1.2 g of blue solid products with yield of 29.7%. ^1H NMR (500 MHz, $CDCl_3$, δ): 8.50 (s, 4H), 8.16 (s, 8H), 7.93 (d, J = 3.4 Hz, 8H), 7.84 (d, J = 3.4 Hz, 4H), 7.65 (d, J = 3.4 Hz, 8H), 7.50 (d, J = 3.4 Hz, 4H), 7.49 (d, J = 3.4 Hz, 8H), 7.45 ~ 7.42 (m, 10H), 7.37 (d, J = 3.4 Hz, 2H), 6.79 (d, J = 3.4 Hz, 2H), 5.94 (s, 2H), 4.39 (t, J = 2.8 Hz, 4H), 3.88 (Br, 4H), 1.97 ~ 1.76 (m, 4H), 1.76 ~ 1.70 (m, 16H), 1.48 ~ 1.25 (m, 112 H); ^{13}C NMR (125 MHz, $CDCl_3$, δ): 180.26, 169.45, 144.25, 142.82, 141.53, 140.67, 140.61, 139.32, 136.60, 131.65, 130.72, 128.44, 127.04, 125.74, 125.38, 123.65, 123.39, 119.04, 116.64, 116.29, 110.70, 109.35, 87.11, 49.38, 43.87, 43.50, 34.79, 32.09, 29.54, 29.50, 29.48, 29.36, 29.19, 27.44, 27.08, 27.00; MALDI-TOF-MS (m/z): 2632.3 (M^+).

General procedure for the synthesis of PFSqGn-*x* polymers.[4]

A mixture of 9,9-dioctylfluorene-2,7-diboronic acid pinacol ester, 2,7-dibromo-9,9-dioctylfluorene and corresponding squaraine-containing co-monomers with corresponding feed ratios, Aliquat 336 (1 drop), $Pd(PPh_3)_4$ (11 mg, 0.01 mmol), 2 M aqueous K_2CO_3 (2 mL), toluene (6 mL) was added and degassed 5 times under nitrogen gas. The resulting mixture was stirred at 100 °C for 48 h, and then end-capped with 0.1 M phenylboronic acid (1 mL) and bromobenzene (1 mL). After cooling, the reaction mixture was poured into methanol and filtered. The precipitate was collected and dissolved in DCM, washed with water and dried with anhydrous Na_2SO4. After evaporating most of the solvent, the residue was precipitated in stirred methanol solvent to give a fiber-like solid. The final product was obtained after drying in vacuum with a yield of 61%–78%.

PFSqG0-1%: Mn = 1.8 x 10^4 Da; PDI = 1.8

PFSqG0-5%: Mn = 1.9 x 10^4 Da; PDI = 2.1

PFSqG1-1%: Mn = 2.1 x 10^4 Da; PDI = 1.9

PFSqG1-5%: Mn = 2.3 x 10^4 Da; PDI = 2.0

PFSqG2-1%: Mn = 4.2 x 10^4 Da; PDI = 2.3

PFSqG2-3%: Mn = 3.5 x 10^4 Da; PDI = 2.4

PFSqG2-5%: Mn = 3.0 x 10^4 Da; PDI = 2.1

PFSqG2-7%: Mn = 3.4 x 10^4 Da; PDI = 2.4

2. Preparations of PFSqGn-*x* Pdots

PFSqGn-*x* Pdots were prepared by the nano-precipitation method according to our previous report.[3] Briefly, the fluorescent semiconducting polymer **PFSqGn-*x*** was dissolved in THF to make a 1 g L^{-1} stock solution first. Then, the stock **PFSqGn-*x*** solution was dissolved in THF with 0.02 g L^{-1} PS-PEG-COOH copolymer to produce a solution mixture with the final **PFSqGn-*x*** concentration of 0.1 g L^{-1}. A 5 mL aliquot of the solution mixture was quickly injected into 10 mL of Milli-Q water under sonication. THF was removed by blowing nitrogen gas into solution at 70 ^oC for about 1 hour. The obtained Pdots solution was sonicated for 1-2 minutes and filtered through a 0.2-μm cellulose membrane filter to remove any aggregates.

3. Materials

Sodium phosphate dibasic (Na_2HPO_4), Sodium phosphate monobasic (NaH_2PO_4), 3-(4,5-Dimethyl-2-thiazolyl)-2,5-diphenyl-2H-tetrazolium bromide (MTT) were purchased from Sigma-Aldrich. 5-(and-6)-Carboxy SNARF®-1 Acetoxymethyl Ester Acetate, Intracellular pH Calibration Buffer Kit, LysoTracker® Green DND-26 - Special Packaging were obtained from ThermoFisher Scientific. Eagle's minimum essential medium (EMEM), fetal bovine serum (FBS), penicillin and streptomycin, were obtained from Am. Type Culture Col. (ATCC). All other reagents were of analytical reagent grade, and used as received. All aqueous solutions were prepared in Milli-Q water (18.2 MΩ cm, Milli-Q, Millipore).

4. Apparatus and characterizations

The ^{1}H NMR and ^{13}C NMR spectra were recorded on Bruker AV 300 or AV500 spectrometers. The small molecular mass spectra were measured using a Bruker Esquire-LC mass spectrometer (ESI-MS) with ion trap messages or Bruker AutoFlex II Matrix-Assisted LASER Desorption Ionization - Time of Flight Mass Spectrometer (MALDI-TOF-MS) for SqG2 monomer. The molecular weight of polymers was determined by gel permeation chromatography using a SHIMADZU LC-20AD liquid chromatograph instrument with polystyrene as standard. The particle size of the Pdots in aqueous solution was characterized by dynamic light scattering (Malvern Zetasizer NanoS). High-resolution Transmission electron microscopic (TEM) measurements were recorded on a FEI Tecnai F20 transmission electron microscope operating at 200 kV. UV-Vis absorption spectra were recorded with DU 720 scanning spectrophotometer (Beckman Coulter, Inc., CA USA). Fluorescence spectra of the Pdots and SNARF-1 in aqueous solution were obtained and calibrated using a commercial fluorimeter (Fluorolog-3, HORIBA Jobin Yvon, NJ USA) and its software with a corrected SOP for NIR emission using a 1 x 1 cm quartz cuvette. Photostability of Pdots in aqueous solution were recorded using the same instrument and a 0.2 x 1 cm quartz cuvette with a maximum volume capacity of 0.6 mL. Fluorescence quantum yields were obtained using a Hamamatsu photonic multichannel analyzer C10027 equipped with CCD and integrating sphere using DI water solvent as the reference. The accurate pH values were measured by Fisher Scientific™ Accumet™ AB15+ Basic and BioBasic™ pH/mV/°C Meters. The fluorescence images of stained cells were acquired with a fluorescence confocal microscope (Zeiss LSM 510).

5. Cell viability

The toxicity of PFSqG$_2$-5% Pdots to cells was measured by the MTT assay. Briefly, MCF-7 cells were plated at a density of 1×10^4 cells per well in 100 μL of EMEM medium in 96-well plates and cultured at 37 ^oC for 24h. The cells then were exposed to a series of concentrations of Pdots for 24h, and the viability of the cells was measured using the MTT method. Controls were cultivated under the same conditions without the addition of the nanocomposites. Then, 100 μL of MTT (0.5 g L^{-1} in EMEM) was added into the wells and further incubated for an additional 4 h. Subsequently, the supernatant was discarded, followed by the additional of 100 μL DMSO into each well to dissolve the formed formazan and incubation in the shaker incubator with gentle shakes for 10 min. Then, the optical density (OD) was read at a wavelength of 570 nm. The cell viability rate was expressed as follow: % = [OD] test/[OD] control× 100%. The cell survival rate from the control group was considered to be 100%.

6. Absorption and Fluorescence spectra measurement

A series of standard pH-PBS buffered solutions were prepared by mixing 10 mM Na_2HPO_4 and 10 mM NaH_2PO_4 at varied volume ratios to obtain the specific and accurate pH values that ranged from 4.7 to 9.52. Then the concentrated Pdots stock solution was dissolved into the above PBS solutions and the final concentration of Pdots was adjusted to 0.01 g L^{-1}. The resulting solution was mixed well and transferred to a quartz cell of 1 cm optical length to measure absorbance against the corresponding blank reagent or fluorescence with the excitation wavelength of λ_{ex} = 400 nm. The ratiometric fluorescence signal (I_{700nm}/I_{438nm}) was calculated from the fluorescence intensities at 700 nm and 438 nm, respectively.

To investigate the fluorescence reversibility of Pdots with pH, the pH of **PFSqGn-*x*** Pdots (0.01 g L^{-1}) solution between pH 5.0 and pH 9.0 was adjusted back and forth by adding 1 M HNO_3 or NaOH with the monitoring by a pH meter. The fluorescence spectra were recorded at λ_{ex} = 400 nm to obtain the ratiometric fluorescence signal.

7. Cell culture and labeling

The breast cancer cell line MCF-7 was obtained from American Type Culture Collection (ATCC, Manassas, VA, USA). Primary cultured MCF-7 cells were cultured in EMEM supplemented with 10% FBS, 1% penicillin and streptomycin and the cells were maintained at 37 ^oC in a humidified atmosphere (95% air and 5% CO_2). Cells were collected at the exponential phase of growth. The adherent MCF-7 cells were harvested from the culture flask by quickly rinsing with fresh culture media followed by incubation with 5 mL of Trypsin-EDTA

solution (0.25 w/v % trypsin, 2.5 g/L EDTA) at 37 ^{o}C for 5 min. After complete detachment, the solution of suspended cells was centrifuged to precipitate the cells. Then 1 × 10^5 MCF-7 cells were dispensed on a glass-bottomed culture dish and allowed to grow for 24 h with 0.01 g L^{-1} Pdots. Prior to fluorescence imaging, the stained cells were rinsed with PBS solution to remove any nonspecifically adsorbed Pdots on cell membrane.

For intracellular pH calibration of MCF-7 cells, PFSqG2-5% Pdots-loaded cells were incubated at 37 ^{o}C for 15 min in intracellular pH calibration buffer of various pH values (pH 5.5~8) in the presence of 10 μM nigericin. Then fluorescence imaging experiments were performed on confocal microscope with excitation at 405 nm through 40X/oil objective. Fluorescence emissions were collected through the following two channels of BP 420-480 (BP means "band pass", anything between the wavelengths indicated can pass through) and LP 650 (LP means "long pass", anything longer than the wavelength indicated can pass through) for blue and NIR, respectively. Image processing and analysis was carried out on Image J software and the ratio of NIR channel to blue channel was calculated pixel-by-pixel for the region of interest (ROI). The cells were maintained at 37 ºC in a humidified atmosphere (95% air and 5% CO_2).

To explore the intracellular pH fluctuation associated with H_2O_2 and NH_4Cl, PFSqG2-5% Pdots-loaded MCF-7 cells first were treated with H_2O_2 or NH_4Cl at 37 ^{o}C for 1 h in PBS solution (pH 7.4) and then subjected to fluorescence imaging.

To perform the co-localization fluorescence imaging, the MCF-7 cells loaded with PFSqG2-5% Pdots were incubated with the pre-warmed (37 ^{o}C) probe (LysoTracker Green DND-26, 50 nM)-containing medium for 5 min. Then the loading solution was replaced with fresh medium and the cells were observed using fluorescence microscope fitted with the proper filter set. Fluorescence imaging experiments were carried out with excitation at either 405 nm (for Pdots) and 488 nm (for Lysotracker) through a 63X/1.2 oil objective. The corresponding fluorescence emissions were collected at BP 420-480 (blue channel of Pdots), LP 650 (NIR channel of Pdots) and BP 505-550 (Green channel of Lysotracker), respectively.

For the comparative study with SNARF-1 acetoxymethyl ester, loading of the SNARF-1 probe into MCF-7 cells was achieved by incubating the pre-cultured cells (cultured for 24h on glass-bottomed culture dish) in EMEM medium containing 10 μM SNARF-1, 0.01% Pluronic F-127 and 0.1% DMSO for 30 min in dark at 21-24 ^{o}C. The intracellular pH calibration and pH fluctuation studies were performed according to the same procedure of Pdots part except that the fluorescence was collected at λex = 488 nm with two channels of BP 530-600 and LP 615, respectively. The images of the ratio channel were obtained via pixel-by-pixel calculation using Image J software.

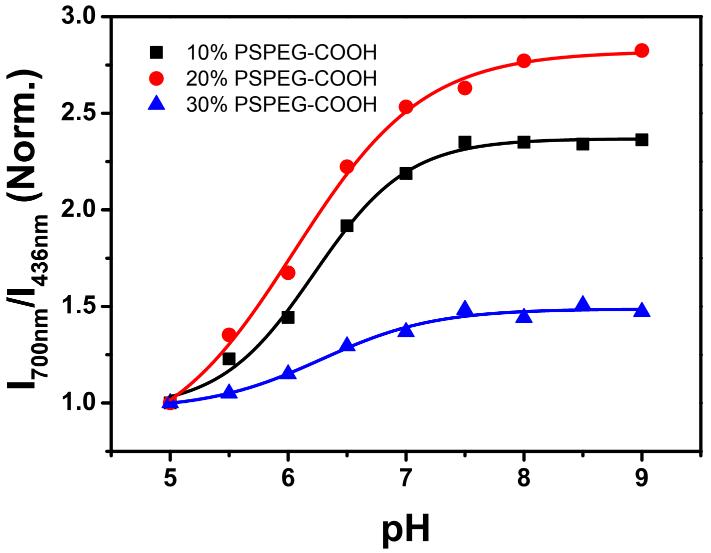

Figure S1. The pH-response curves of PFSqG2-5% Pdots with different PS-PEG-COOH content in HEPES buffer.

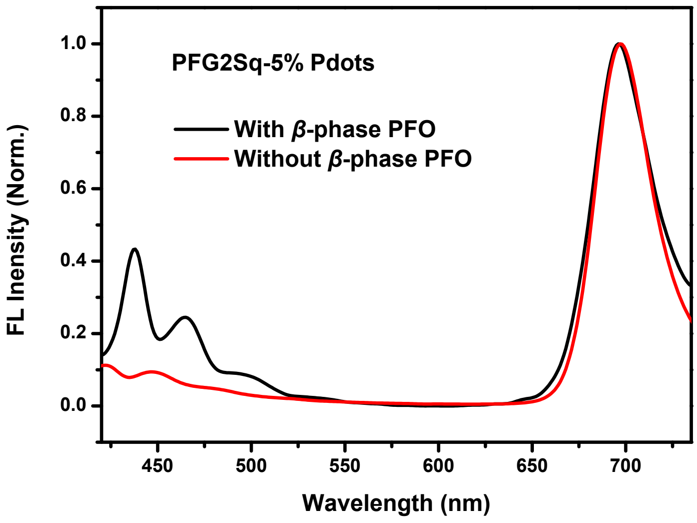

Figure S2. The PL spectra of PFG2Sq-5% Pdots with and without *β*-phase PFO.

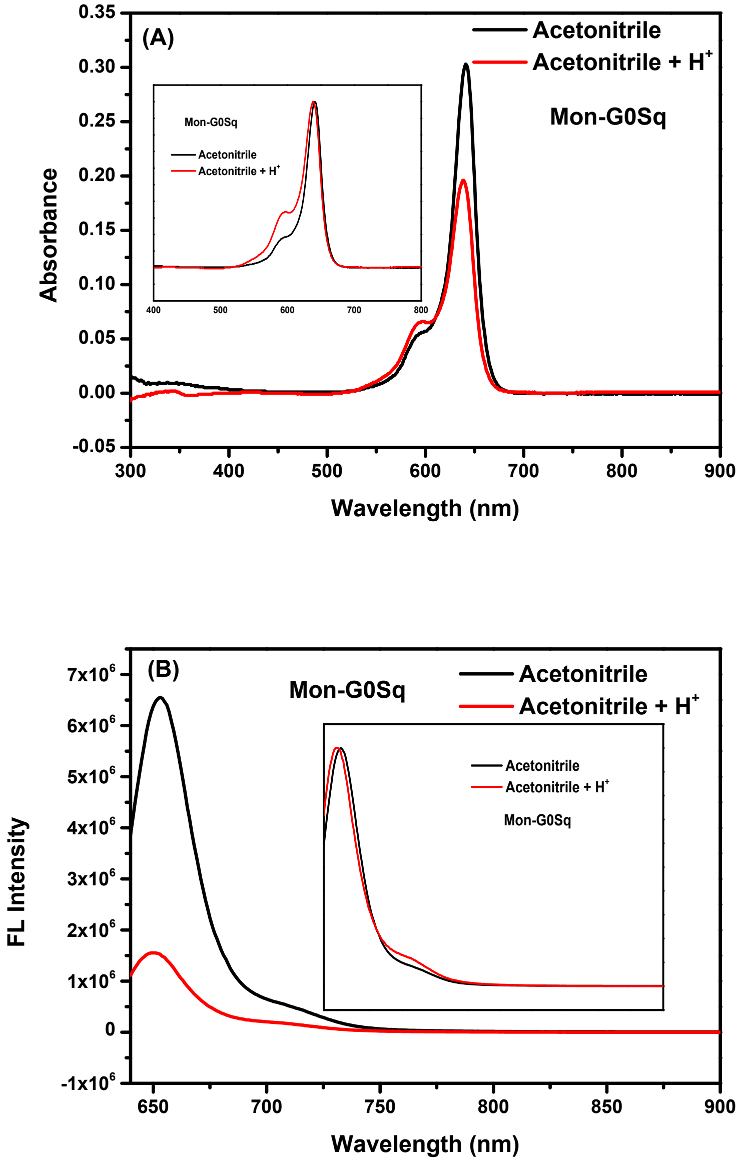

Figure S3. Absorption (A) and FL spectra (B) of G0Sq monomer in acetonitrile and acetonitrile with 2 vol% 6 M HCl.

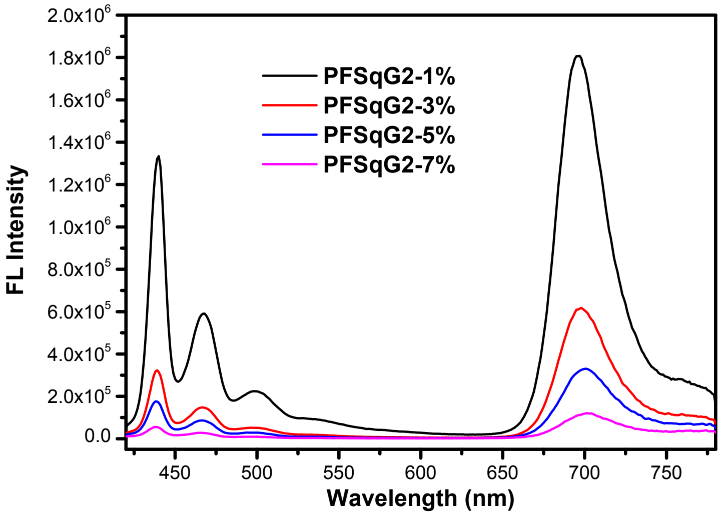

Figure S4. Fluorescence (FL) spectra of 0.1 g L^{-1} PFSqG2-*x* Pdots in DI water.

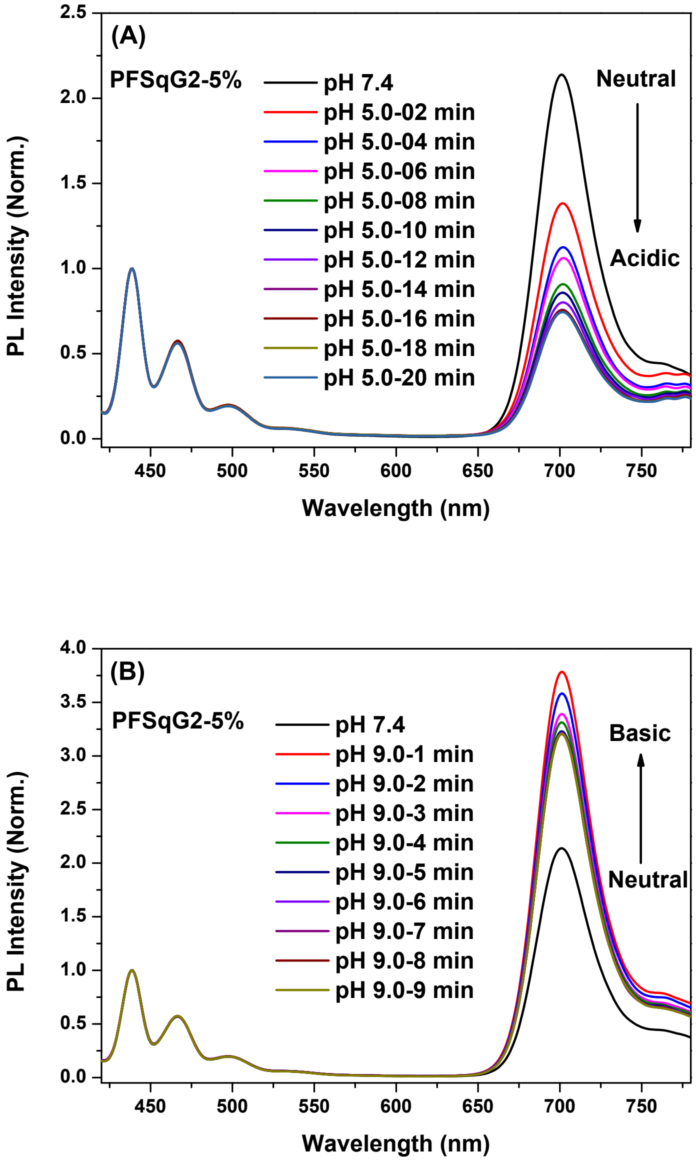

Figure S5. The normalized time dependent fluorescence (FL) spectra from neutral to acidic (A) and basic (B) pH.

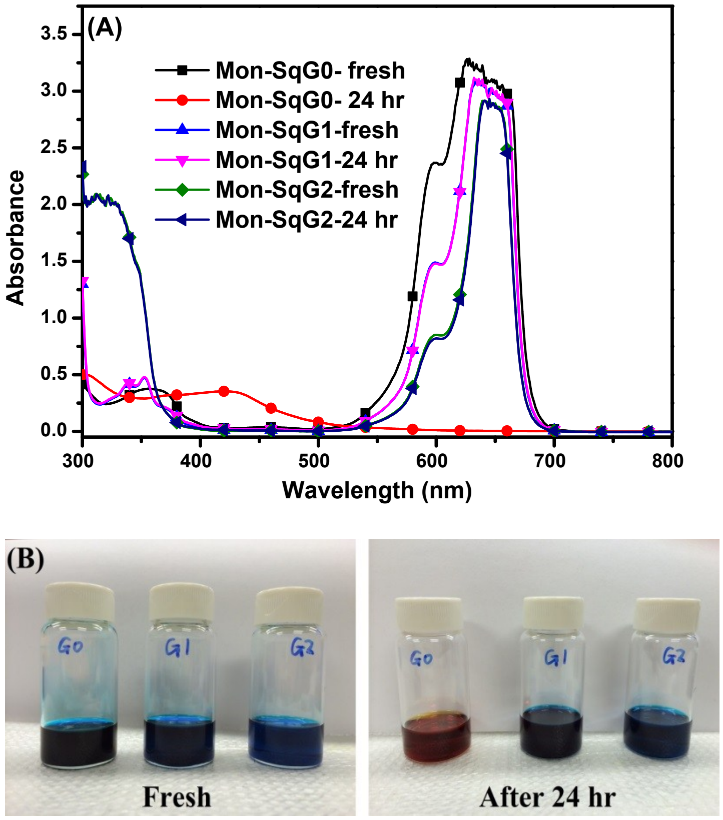

Figure S6. Absorption spectra (A) and corresponding pictures (B) of 0.1 g L^{-1} Monomer-SqGn in fresh THF solution and after standing 24 hr in ambient light.

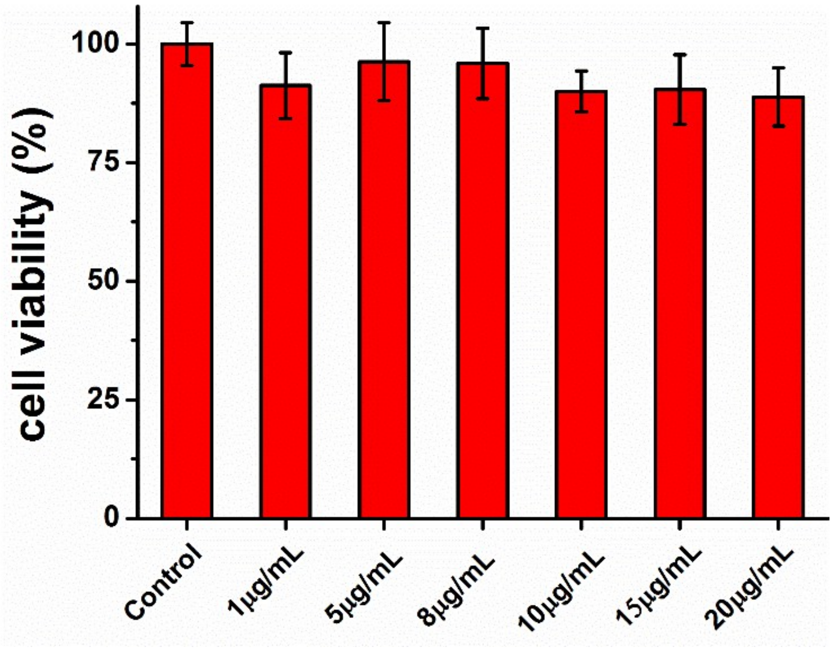

Figure S7. MTT assay of PFSqG2-5% Pdots.

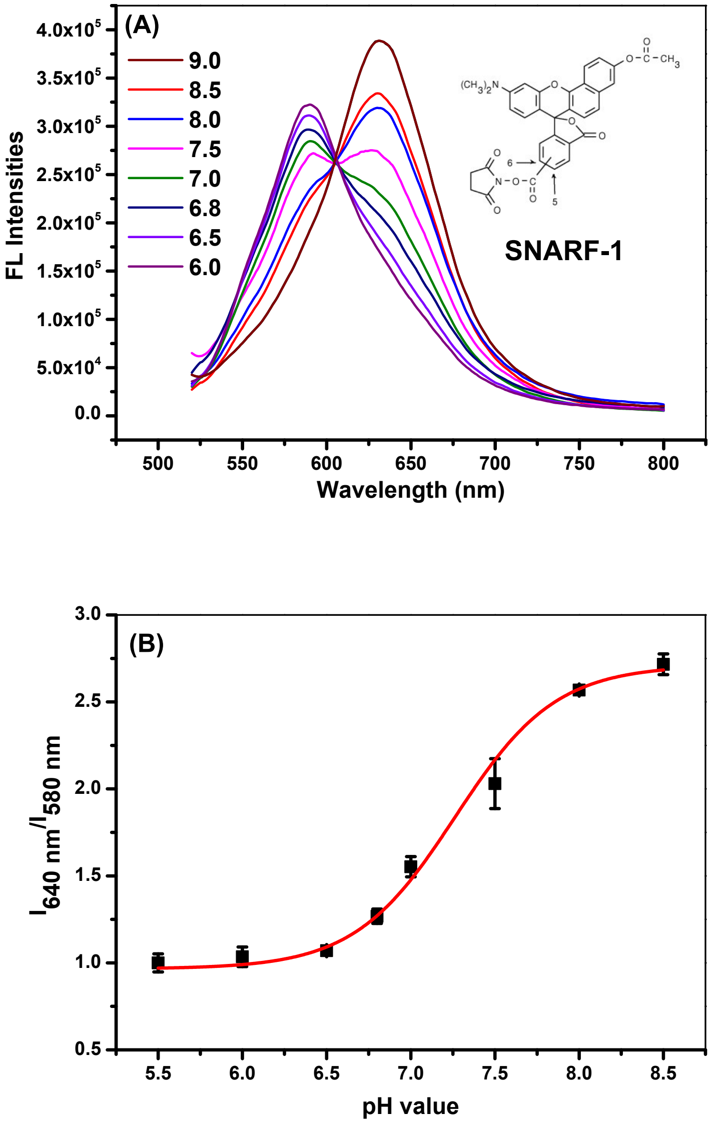

Figure S8. Emission spectra of SNARF-1 in PBS solution with different pH values (A) and the plot of normalized I640nm/I580nm obtained from SNARF-1 (B).

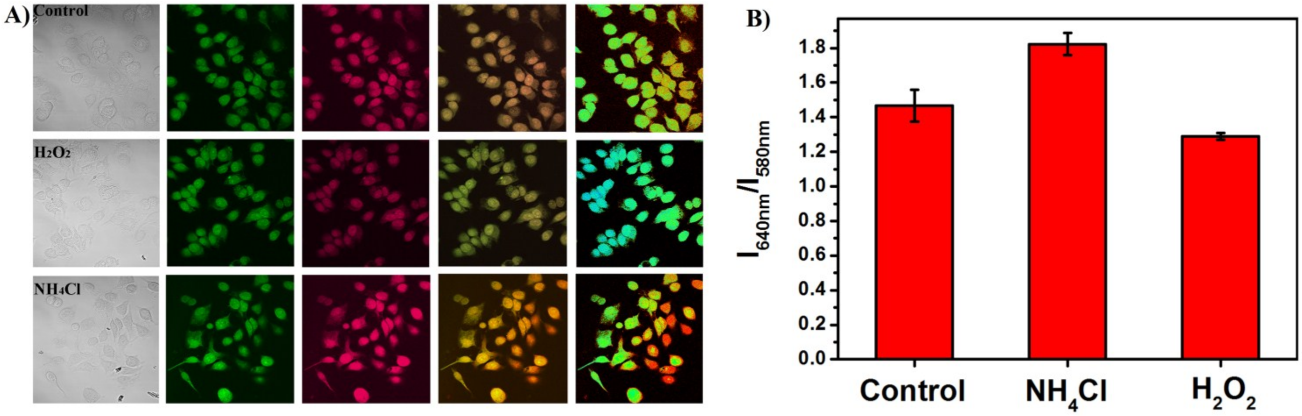

Figure S9. The impact of NH_4Cl and H_2O_2 on intracellular pH measured by SNARF-1. Intracellular pH of cells treated with NH_4Cl and H_2O_2 in pH 7.4 PBS buffer solution (A); Calculated ratio of $I_{640\ nm}/I_{580\ nm}$ of SNARF-1 probe for the control and treated cells (B)

Refences

1 X. He, L. Chen, Y. Zhao, S. C. Ng, X. Wang, X. Sun and X. Hu, *RSC Adv.* **2015**, *5*, 15399.

2 S. F. Völker, S. Uemura, M. Limpinsel, M. Mingebach, C. Deibel, V. Dyakonov and C. Lambert, *Macromol. Chem. Phys.* **2010**, *211*, 1098.

3 I-C. Wu, J. Yu, F. Ye, Y. Rong, M. E. Gallina, B. S. Fujimoto, Y. Zhang, Y-H. Chan, W. Sun, X. H. Zhou, C. Wu and D. T. Chiu, *J. Am. Chem. Soc.* **2015**, *137*, 173.

4 L. Chen, B. Zhang, Y. Cheng, Z. Xie, L. Wang, X. Jing and F. Wang, *Adv. Funct. Mater.* **2010**, *20*, 3143.
